# Overexpression of herbaceous peony *HSP70* confers high temperature tolerance

**DOI:** 10.1186/s12864-019-5448-0

**Published:** 2019-01-21

**Authors:** Daqiu Zhao, Xing Xia, Jianghong Su, Mengran Wei, Yanqing Wu, Jun Tao

**Affiliations:** Jiangsu Key Laboratory of Crop Genetics and Physiology, College of Horticulture and Plant Protection, Yangzhou University, Yangzhou, 225009 People’s Republic of China

**Keywords:** Heat shock proteins, Reactive oxygen species, High temperature, Stress, Herbaceous peony

## Abstract

**Background:**

Heat shock proteins (HSPs) are found extensively in Eukaryotes and are involved in stress tolerance. However, their functions in herbaceous peony (*Paeonia lactiflora* Pall.) under high temperature stress are poorly characterized.

**Results:**

In this study, the genomic sequence of *P. lactiflora HSP70*, designated *PlHSP70*, was isolated. Its full-length was 3635 bp, and it contained a large 1440-bp intron. The encoded protein with a molecular weight of 71 kDa was localized in the cytoplasm of the cell. *PlHSP70* transcription was detected in *P. lactiflora* and increased with the treatment of high temperature stress. The constitutive overexpression of *PlHSP70* in *Arabidopsis thaliana* obviously conferred tolerance to high temperature stress by affecting different physiological and biochemical indices. Transgenic *A. thaliana* plants exhibited higher chlorophyll fluorescence values than the wild-type (WT) when exposed to high temperature stress. The accumulation of hydrogen peroxide (H_2_O_2_), superoxide anion free radical (O_2_^·-^) and relative electric conductivity (REC) were significantly lower in the transgenic *A. thaliana* plants compared to the WT. In addition, more intact cell membranes, chloroplasts and starch grains, and fewer plastoglobuli were found in the *PlHSP70*-overexpressing transgenic lines than in the WT.

**Conclusions:**

All of these results indicated that *PlHSP70* possessed the ability to improve the tolerance to high temperature in transgenic *A. thaliana*, which could provide a theoretical basis to improve high temperature tolerance of *P. lactiflora* by future genetic manipulation.

**Electronic supplementary material:**

The online version of this article (10.1186/s12864-019-5448-0) contains supplementary material, which is available to authorized users.

## Background

Plant growth and development is often affected by all types of stresses, including biotic and abiotic stresses. Abiotic stresses such as drought, salinity, and extreme temperature are important factors limiting the growth of plants [1]. In the context of global warming and the increased frequency of extreme temperature, high temperature has been well recognized as a highly significant environmental factor that represents a serious threat to almost all aspects of plant development, growth, reproduction, and yield worldwide [2]. For example, the germination rate index of lima bean (*Phaseolus lunatus* L.) seeds under 35 °C was significantly lower than that at 25 °C and 30 °C [3]. In addition, the floret pollination and fertilization in rice (*Oryza sativa* L.) could not be conducted normally during the heading and flowering period above 35 °C, and the fertilization rate decreased significantly [4, 5]. In addition, wheat (*Triticum aestivum* L.) exposure to high temperature significantly decreased the rate and duration of grain filling and decreased the grain weight [6]. To prevent the damage caused by high temperature, plants developed various defence mechanisms, including a complex metabolic adjustment process known as the heat shock response [2].

The induced synthesis of heat shock proteins (HSPs) is a major component of the heat shock response, which can decrease the harmful effects of high temperature. HSPs are a class of the most ubiquitous and stress-resistant protective proteins which primarily act as molecular chaperones and play a crucial role in protecting the cells from oxidative damage through the folding and translocation of newly translated proteins and the repair of misfolded proteins to maintain the stability of the internal environment of the plant [7, 8]. Based on their apparent molecular mass, plant HSPs are divided into five classes: HSP100, HSP90, HSP70, HSP60 and small heat shock proteins (sHSP) [9]. HSP70 is the most abundant and highly conserved of the five classes in most organisms and acts as a molecular chaperone, and it is currently the most highly studied heat shock protein [10]. When exposed to high temperature treatment at 35–42 °C for 12–168 h, the HSP70 content was up-regulated in cucumber (*Cucumis sativus* L.) [11], pepper (*Capsicum annuum* L.) [12], lettuce (*Lactuca sativa* L.) [13], luffa (*Luffa cylindrica* (L.) Roem.) [14] and tomato (*Solanum lycopersicum* L.) [15]. In addition, the overexpression of non-heading Chinese cabbage (*Brassica campestris ssp. chinensis* Makino) *HSP70* confers high temperature tolerance to tobacco [16], and the introduction of *Porphyra seriata HSP70* into *Chlamydomonas* can effectively enhance its resistance to high temperature stress [17]. Therefore, *HSP70* plays a crucial role in helping to protect plants from high temperature damage, but there has been little in-depth study on this topic in ornamental plants.

As one of the traditional flowers of China, herbaceous peony (*Paeonia lactiflora* Pall.) has a broad market prospect because of its large, multicolored and beautiful flowers. It could be used as potted flowers, cutting flowers and materials for gardening to make unique seasonal landscapes [18]. However, the damage caused by high temperature limits the popularization and application of *P. lactiflora*. High temperature in the summer results in the yellowing and withering of the *P. lactiflora* leaves resulting in dead spots, particularly in the middle and lower reaches of the Yangtze River to southern China. Plant growth vigour is excessively reduced, and diseases and insect pests are serious problems, which severely affect its beauty and the growth during the following year. But in this field, only several studies had preliminarily clarified its biochemical and molecular responses [19–21], and these studies all found that *HSP70* play a critical role in the resistance of *P. lactiflora* to high temperature. However, more in-depth studies to validate the function of the high temperature resistance of *P. lactiflora HSP70* have not been performed. In this study, we isolated the genomic sequence of *P. lactiflora HSP70* and studied its heterologous expression in *Escherichia coli*, subcellular localization in *O. sativa* protoplasts and expression patterns in *P. lactiflora* with high temperature stress treatment. In addition, we generated transgenic *Arabidopsis thaliana* plants that overexpressed *P. lactiflora HSP70* to compare the high temperature tolerance and systematically studied its underlying mechanism. These results could provide a theoretical basis to improve the high temperature tolerance of *P. lactiflora* by genetic manipulation in the future.

## Methods

### Isolation and bioinformatics analysis of *PlHSP70* genomic sequence

According to the full-length cDNA sequence of *PlHSP70* (accession number in NCBI: JN180465), gene-specific primers (forward primer: 5’-CTCTTACTTTTCTTCTCTCGACCC CTTCCG-3′, reverse primer: 5’-CTCTTACTTTTCTTCTCTCGACCCCTTCCG-3′) were designed to isolate its genomic DNA sequence. Total DNA extraction was performed according to MiniBEST Plant Genomic DNA Extraction Kit (TaKaRa, Japan). The extracted total DNA was used as a template for polymerase chain reaction (PCR) to obtain the genomic DNA sequence of *PlHSP70*. The genomic DNA sequence was amplified in a total volume of 25 μL reaction system containing total DNA 2 μL, 10 × PCR Buffer 2.5 μL, dNTP Mixture (2.5 mM each) 2 μL, TaKaRa Taq™ (5 u/μL) 0.2 μL (TaKaRa, Japan), PCR primers (10 μM) (Additional file 1: Table S1) 2.5 μL and ddH_2_O 15.8 μL. PCR conditions were 3 min at 94 °C, followed by 35 cycles of 30 s at 94 °C, 30 s at 51 °C, and 240 s at 72 °C, with a final extension at 72 °C for 10 min. PCR products were separated by 1% agarose gel electrophoresis and sent to Shanghai Sangon Biological Engineering Technology & Services Co., Ltd. (Shanghai, China) for sequencing. Sequence comparison was performed using DNAMAN 5.2.2.

### Heterologous expression of *PlHSP70* in *E. coli*

The expression plasmid of *PlHSP70* was constructed. The full-length of *PlHSP70* was amplified with primers that included *Not*I/*Cpo*I restriction sites (forward 5’-GATCCGGTCCGAAACTCTTACTTTTCTTCTCTCGACCC-3′, reverse 5’-TGCAGCGGCCGCTTAGTCGACCTCTTCAATCTTGGGA-3′) and ligated into the *pET-sumo* vector, which was treated with the Champion™ pET SUMO Expression System (Thermo Fisher Scientific, USA). The ligation (*pET-PlHSP70-sumo*) mixture was chemically transformed into the *E. coli* Top 10 competent cells. The recombinant cells were plated on LB medium (50 μg/mL Kanamycin sulfate) and grown at 37 °C for 16 h. Single colony of recombinant plasmid was inoculated into 3 mL LB (50 μg/mL Kanamycin sulfate) with the same condition. At an OD_600_ of 0.6, IPTG was added to a final concentration of 0.1 mM. The cells were further grown at 37 °C for another 3 h. After centrifugation at 12,000×g for 120 s, the pellet was harvested and resuspended in 40 μL washing buffer (0.3 M NaCl, 20 mM imidazole, 50 mM Tris-HCl, pH 8.0). Lysozyme was added to the suspension at a final concentration of 1 mg/mL. Then, the mixture was incubated at 37 °C for 30 min and inserted into an ice bath for 15 min. Ultrasonication of the samples was then performed on ice with a 40% duty cycle, pulse on/off 9.9 × 10 s pause. The disrupted cells were centrifuged at 8,000×g for 30 min and the supernatant was collected. A HiTrap™ chelating HP column (Amersham Biosciences, USA) was used to purify *PlHSP70* protein from *E. coli*. After equilibrated with washing buffer, the cell-free extract was loaded onto the column and washed with 150 mL washing buffer. The isocratic elution consisted of eluting buffer (0.3 M NaCl, 50 mM Tris-HCl, 100 mM imidazole, pH 8.0) at 1 mL/min flow rate. The 2.5 mL fractions were collected and analysed by sodium dodecyl sulfate polyacrylamide gel electrophoresis (SDS-PAGE) to visualise the *PlHSP70* protein.

### Subcellular localization of *PlHSP70*

Subcellular localization of *PlHSP70* was determined using confocal laser microscopy in *O. sativa* protoplasts. *PlHSP70* product was obtained by constructing gene fusions of *p35S::PlHSP70*-*GFP*. The open reading frame (ORF) of *PlHSP70* was amplified with primers that included *Bsa*I/*Esp*3I restriction sites (forward 5’-CAGTCGTCTCACAACATGGCAGGCAAAGGAGAAGG-3′, reverse 5’-CAGTCGTCTCATACAGTCGACCTCTTCAATCTTGG-3′), which was digested and the product ligated into the expression vectors with T4 DNA ligase (TaKaRa) to generate a set of *pBWA(V)HS*-*PlHSP70*-*GFP* fusion, subsequently sequenced for verification. The *p35S::PlHSP70*-*GFP* constructs and the empty *pBWA(V)HS*-*GFP* vector were transformed into *O. sativa* protoplasts using a modified procedure that was described previously [22]. Gently mix 200 μL protoplast suspension, 10 μL plasmid DNA and 10 μL marker plasmid DNA together with 220 μL PEG solution, standing for 30 min at room temperature. After concentration at 100×g for 5 min, after which the precipitate was suspended in W5 medium. The transformed protoplasts were then incubated at 28 °C for 24 h in W5 medium in the dark. The transient expression of GFP and mkate were monitored by confocal laser microscopy combination system (LSM510/ConfoCor2, Zeiss, Germany).

### Expression pattern analysis of *PlHSP70* under high temperature stress

The 3-year-old *P. lactiflora* ‘Da Fugui’ in potting soil (loam: peat: coarse sand, 1:1:1) were used for expression pattern analysis of *PlHSP70* under high temperature stress. Plants were grown in a growth chamber at 40 °C on a 14 h light/10 h dark cycle, the light intensity was 30–40 μM/m^2^ s and the relative humidity was 60%. And the leaves were taken on 0, 1, 2 and 3 days after treatment. One part samples were used for relative electric conductivity (REC) determination, and the others were immediately frozen in liquid nitrogen, and then stored at − 80 °C until *PlHSP70* expression pattern analysis.

### Overexpressing *PlHSP70* in Transgenic *A. thaliana*

The ORF sequence of *PlHSP70* gene was amplified with primers that included *BamH*I/*Kpn*I restriction sites (forward 5’-CGCGGATCCATGGCAGGCAAAGGA-3′, reverse 5’-CGGGGTACCTTAGTCCACCTCTTCAA-3′) and ligated into the plant expression vector pCAMBIA1301 behind the cauliflower mosaic virus (CaMV) 35S promoter. The *pCAMBIA1301-PlHSP70* plasmid was introduced into the *Agrobacterium tumefaciens* strain EHA105 via the freeze-thaw method and then transformed into *A. thaliana* using the floral dip method [23]. Seeds from transgenic *A. thaliana* and non-transgenic *A. thaliana* plants (wild-type, WT) were harvested from individual plants and sown again. In order to identify transgenic plants, the screening medium (1/2 MS + 30 g/L sucrose + 6.5 g/L agar + 25 mg/L ampicillin (Amp) + 25 mg/L hygromycin (Hyg), pH 5.8) and GUS staining method were used to screen transgenic *A. thaliana*, and when the screening transgenic *A. thaliana* grew to the bolting stage, leaves were collected for PCR.

Before *A. thaliana* grew to the bolting stage, plants were transferred to a growth chamber at 40 °C in continuous light for 48 h. After high temperature treatment, plants were firstly used for chlorophyll fluorescence parameters measurement, as well as hydrogen peroxide (H_2_O_2_), superoxide anion free radical (O_2_^·-^), REC and anatomy observation, and then were immediately frozen in liquid nitrogen and stored at − 80 °C until further quantitative real-time PCR (qRT-PCR).

### Physiological indices measurement and anatomy observation

H_2_O_2_ accumulation was detected by diaminobenzidine (DAB) staining [24]. Briefly, the *A. thaliana* leaves were immersed in 0.1 mg/mL DAB in 50 mM Tris-acetate buffer, pH 5.0, at 25 °C for 24 h in the dark, and then they were boiled in 95% (*v*/v) ethanol for 15 min, and then photographed using a camera (Canon 50D, Japan). O_2_^·-^ accumulation was detected by a reagent kit (Shanghai Haling Biotechnology Co., Ltd., China). The samples were observed at 540 nm excitation wavelength and 590 nm emanation wavelength, and imaged with a fluorescent microscope (Axio Imager D2, ZEISS, Germany). The fluorescent signals were gathered using ZEN software (ZEISS, Germany). Relative water content of leaf was measured using the oven (Jinghong Laboratory Instrument Co., Ltd., Shanghai, China) and balance (Gandg Testing Instrument Factory, Changshou, China). MDA content was performed according to the guidelines of a reagent kit from Nanjing Jiancheng Bioengineering Institute, China. REC was determined according to the method reported by Yang et al. [25]. Additionally, the chlorophyll fluorescence parameters were measured with a chlorophyll fluorescence spectrometer (Heinz Walz GmbH 91,090 Effeltrich, Germany).

The anatomical details of leaves were observed by the transmission electron microscope (Tecnai 12, Philips, Holland). The fixed leaves were washed 3 times with 0.1 mol/L phosphate buffer for 15 min, and post-fixed with 1% osmium tetroxide for 4 h at room temperature (25 °C). After washing 3 times with 0.1 mol/L phosphate buffer for 15 min each, the leaves were dehydrated using 50, 70, 85, 95 and 100% gradient ethanol for 15 min each. Moreover, they were treated with 100% acetone solution (15 min) and acetone solution containing anhydrous sodium sulfate (15 min), infiltrated in Spurr resin and then hardened at 70 °C for 24 h. Sections (70 nm thick) were cut with a diamond knife using a Leica EM UC6 ultramicrotome (Leica Co., Austria) and stained with 1% uranyl acetate in 70% methanol, and 1% lead citrate before examination. Finally, the samples were observed and imaged.

### PCR and gene expression analysis

To identify the transformed plants, total RNA was extracted from *A. thaliana* leaves using MiniBEST Plant RNA Extraction Kit (TaKaRa, Japan). The cDNA was synthesized from RNA using PrimeScript® RT reagent Kit With gDNA Eraser (TaKaRa, Japan). PCR was performed in a total volume of 25 μL reaction system containing total cDNA 2 μL, 10 × PCR Buffer 2.5 μL, dNTP Mixture (2.5 mM each) 2 μL, TaKaRa Taq™ (5 u/μL) 0.2 μL (TaKaRa, Japan), PCR primers (10 μM) (Additional file 1: Table S1) 2.5 μL, ddH_2_O 15.8 μL. PCR conditions were 3 min at 94 °C, followed by 35 cycles of 30 s at 94 °C, 30 s at 52 °C, and 30 s at 72 °C, with a final extension at 72 °C for 10 min.

Gene transcript levels were analyzed using qRT-PCR with a BIO-RAD CFX Connect™ Optics Module (Bio-Rad, USA), and all gene-specific primers were shown by Additional file 1: Table S1. qRT-PCR was performed using the SYBR® Premix Ex Taq™ (Perfect Real Time) (TaKaRa, Japan) and contained 12.5 μL 2 × SYBR Premix Ex Taq™, 2 μL cDNA solution, 2 μL mix solution of target gene primers and 8.5 μL ddH_2_O in a final volume of 25 μL. The amplification was carried out under the following conditions: 95 °C for 30 s, 40 cycles at 95 °C for 5 s, 51 °C for 30 s, and 72 °C for 30 s. Gene relative expression levels of target genes were calculated by the 2^-△△Ct^ comparative threshold cycle (Ct) method [26]. The Ct values of the triplicate reactions were gathered using the Bio-Rad CFX Manager V1.6.541.1028 software.

### Statistical analysis

All experiments described here were repeated three times arranged in a completely randomized design. Sequences analysis was performed by DNAMAN 5.2.2 software. Primers were designed using a Primer 5.0 program. All data were means of three replicates with standard deviations. The results were analyzed for variance using the SAS/STAT statistical analysis package (version 6.12, SAS Institute, Cary, NC, USA).

## Results

### Cloning and sequence analysis of genomic DNA

**Fig. 1:**
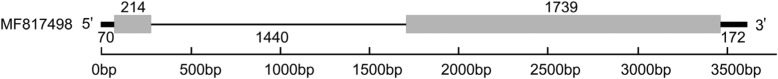
Genomic organization of *PlHSP70*. Exons are represented as grey boxes, introns as thin lines and the untranslated region as bold black lines

The genomic sequence of *PlHSP70* was obtained using PCR method based on its full-length cDNA sequence (JN180465) and extracted DNA. Sequence analysis indicated that the size of the *PlHSP70* genomic DNA was 3635 bp. To elucidate its genomic organization, the genomic DNA and cDNA sequences were aligned using the software DNAMAN 5.2.2, and the results indicated that *PlHSP70* consisted of two exons and one intron, and their sizes were summarized in Fig. 1. This intron began with the GT sequence and ended with AG confirming the consensus 5′ and 3′ intron splice sites for the mRNA. This sequence was deposited in GenBank with accession number MF817498.

### Heterologous expression and subcellular localization of *PlHSP70*

**Fig. 2:**
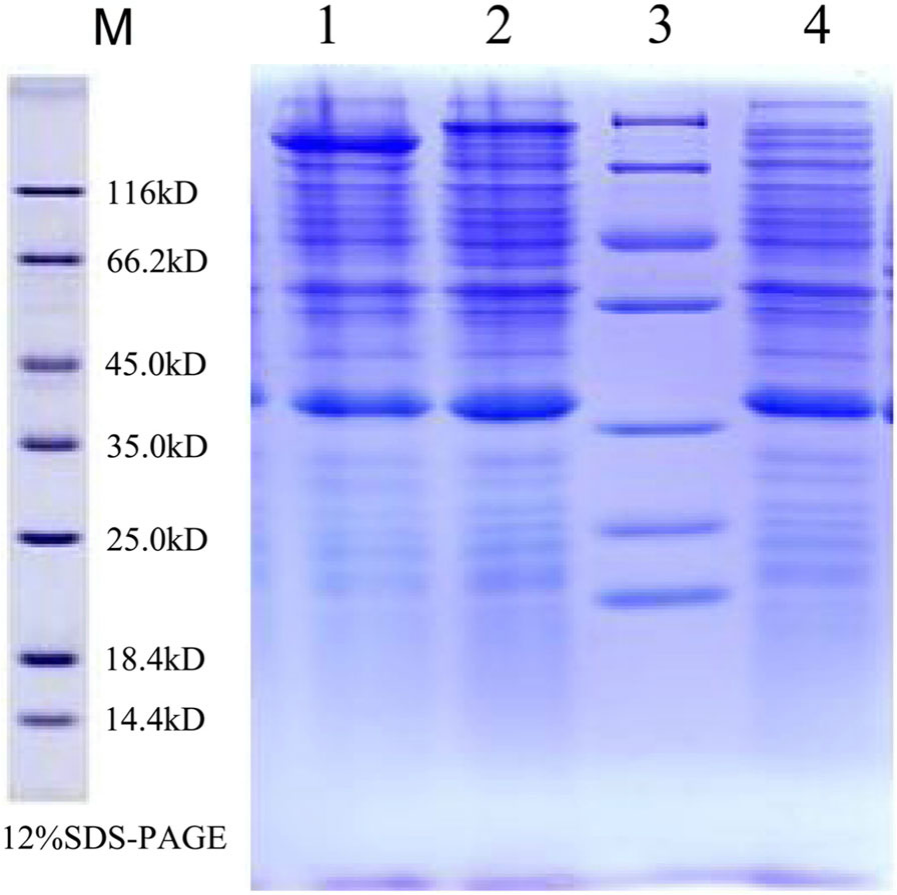
Heterologous expression of *PlHSP70* protein in *E. coli*. M: Protein marker; 1: IPTG induced *pET-PlHSP70-sumo* rose; 2, Non-IPTG-induced soluble fraction of *E. coli pET-PlHSP70-sumo* rose; 3, Protein marker; 4, Empty *pET-sumo rose* vector

The heterologous expression of the recombinant *PlHSP70* plasmid (*pET-PlHSP70-sumo* rose) was constructed and transformed into *E. coli* cells. The recombinant *E. coli* cells were induced using IPTG, the cells were harvested and the protein was isolated. The soluble fractions from the recombinant strains differed from those of the non-IPTG-induced *pET-PlHSP70-sumo* rose and empty *pET-sumo rose* vector, and the apparent molecular weight of the *PlHSP70* protein plus the GST-Tag protein (16 kDa) was approximately 87 kDa, thus, the molecular weight of the *PlHSP70* protein was approximately 71 kDa, which was consistent with its putative molecular weight (Fig. 2).

**Fig. 3:**
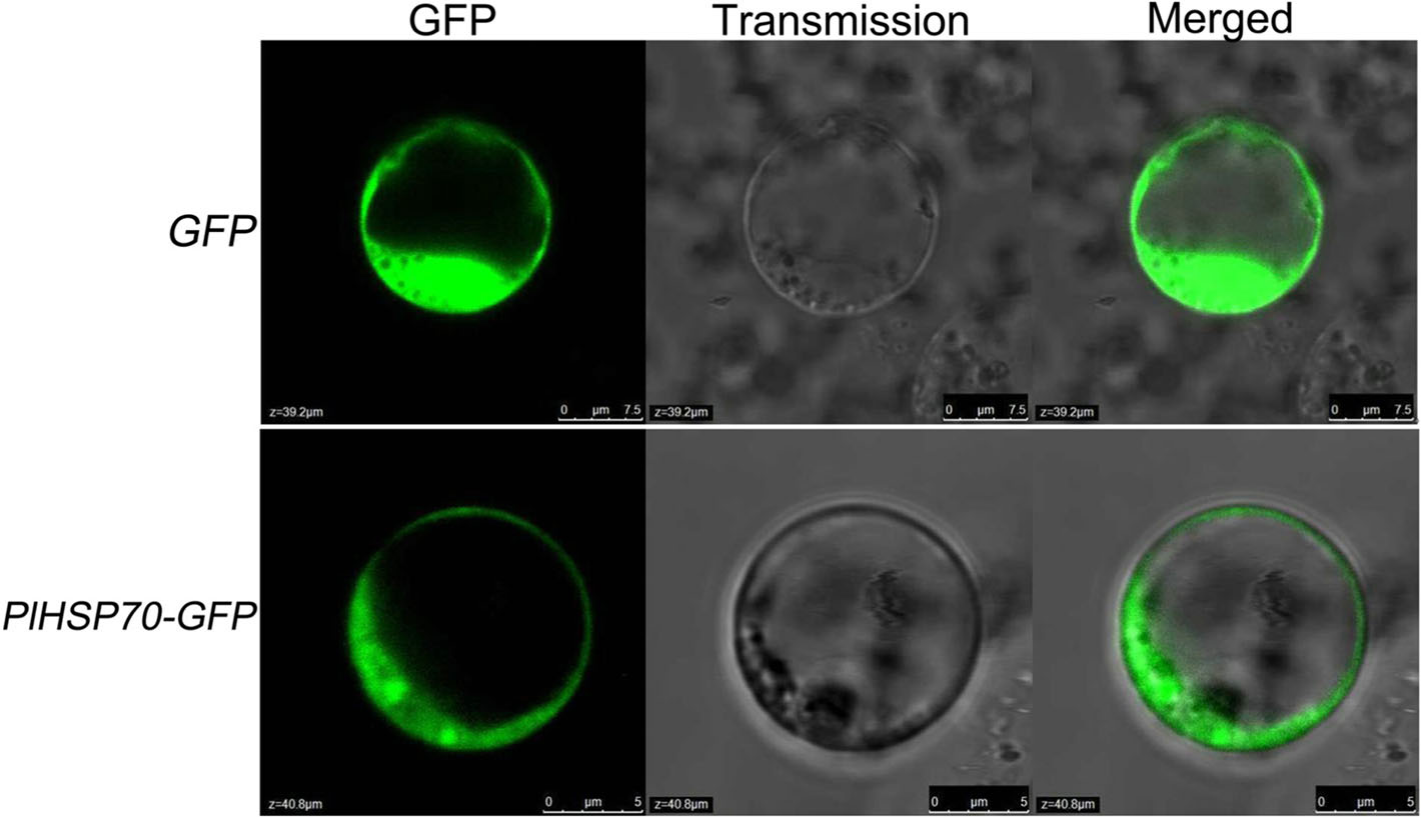
Subcellular localization of *PlHSP70* in rice protoplasts

In addition, the subcellular localization of *PlHSP70* was observed. When transient transformation with *PlHSP70*-*GFP* driven by the 35S promoter occurred in *O. sativa* protoplasts, *PlHSP70*-*GFP* fluorescence indicated that *PlHSP70* was localized in the cytoplasm of the cell (Fig. 3).

### Expression pattern analysis of *PlHSP70* under high temperature stress

**Fig. 4:**
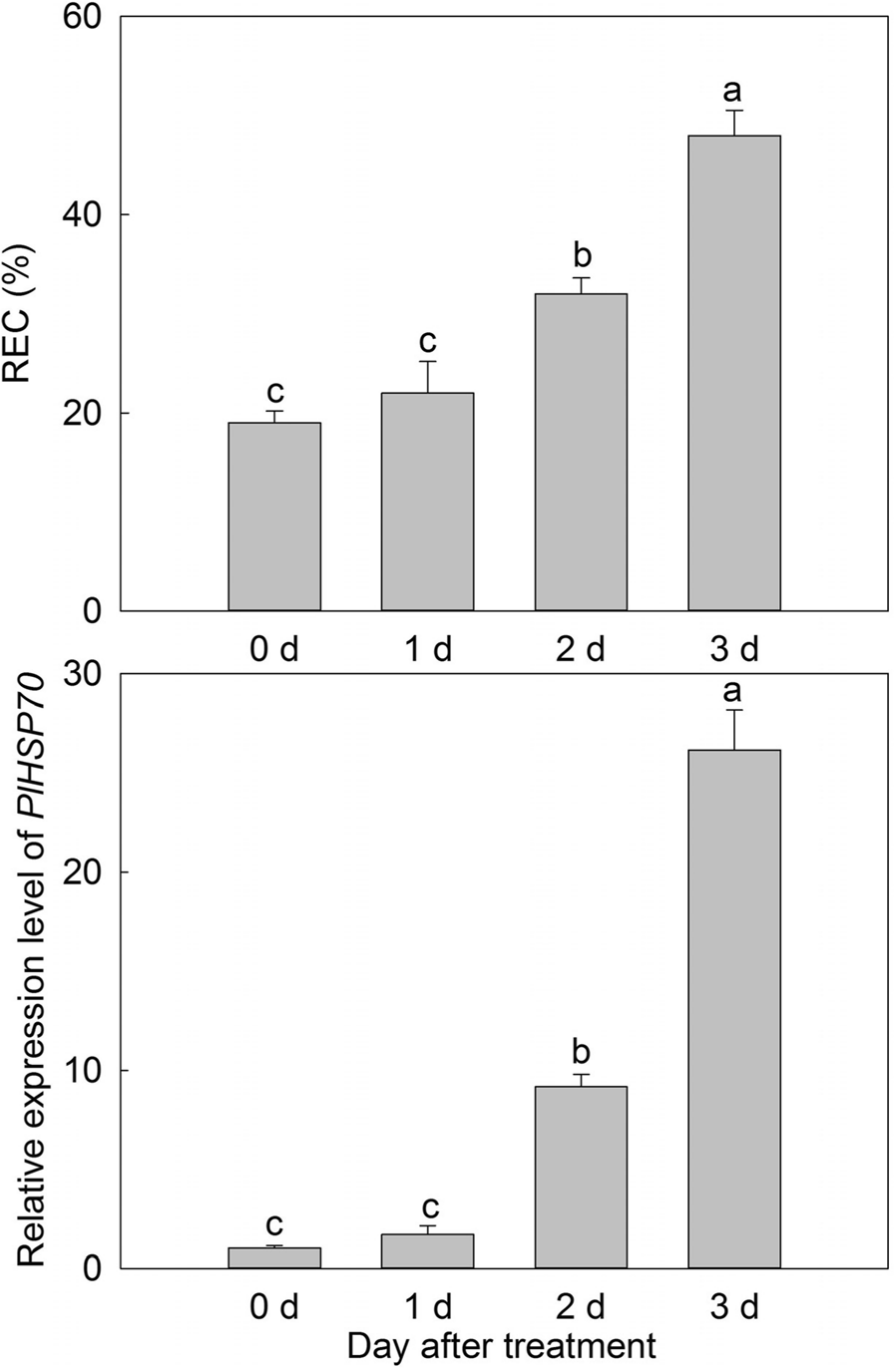
REC and the relative expression level of *PlHSP70* in *P. lactiflora* under high temperature stress. All data are the means of three replicates with standard deviations, and different letters indicate significant differences according to Duncan’s multiple range test (*P* < 0.05)

To examine the expression pattern of *PlHSP70* treated under high temperature stress, *P. lactiflora* ‘Da Fugui’ in potting soil was exposed to a high temperature (40 °C) for 0, 1, 2 and 3 days. And total RNA was isolated from leaves for qRT-PCR with gene-specific primers as described above. Before qRT-PCR, REC reflecting the membrane lipid peroxidation was first measured. It increased with the treatment and peaked on 3 d, and the value of 0 d was only 39.58% of that on 3 d, which revealed that the damage of high temperature to plants became more intense with the treatment. In addition, the relative expression level of *PlHSP70* demonstrated the same tendency, and it was quickly induced on 1 d and peaked on 3 d, resulting in expression levels up to 26-fold of those at 0 d (Fig. 4).

### Transgenic plants identification

**Fig. 5:**
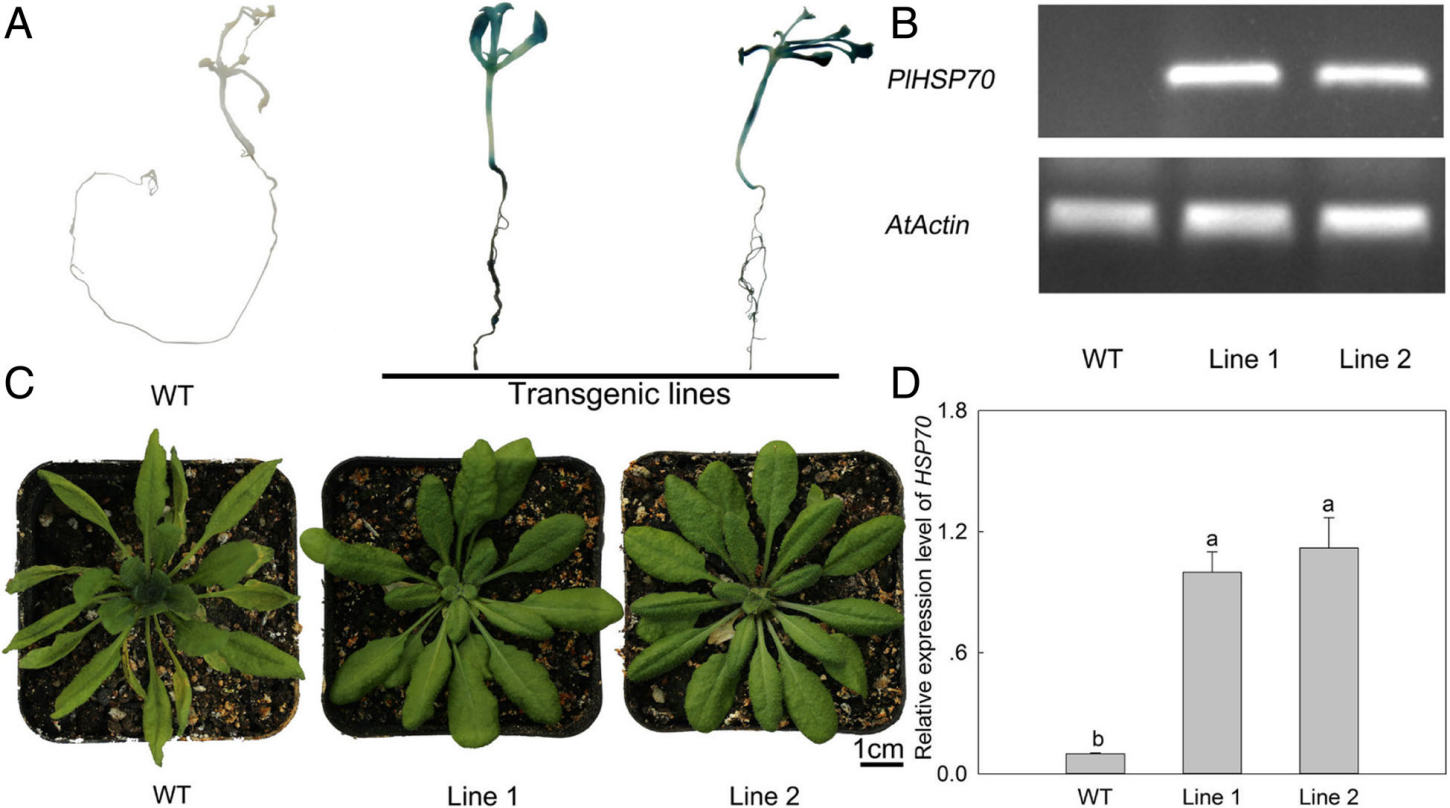
Identification of transgenic *A. thaliana* plants and their phenotype under high temperature stress. **a** GUS staining. **b** PCR analysis of *PlHSP70* mRNA. **c** Phenotype of WT and transgenic lines. **d** Relative expression level of *PlHSP70* in leaves. All data are the means of three replicates with standard deviations, and different letters indicate significant differences according to Duncan’s multiple range test (*P* < 0.05)

To identify the transgenic *A. thaliana* plants, the harvested seeds were first sown on the screening medium with antibiotics. In addition, the identified transgenic plants were randomly selected for GUS staining. As shown in Fig. 5a, the WT remained white, and the transgenic lines all turned blue in the GUS staining buffer. In addition, the DNA extracted from the leaves was used for identification using PCR and the results showed that one clear and bright band could be observed in the *PlHSP70*-overexpressing transgenic lines, while there was no specific band in WT (Fig. 5b). Subsequently, these *A. thaliana* plants were treated with 40 °C for 48 h, and Fig. 5c showed that the leaves of WT turned to yellow, shrunk and tended to die, while the *PlHSP70*-overexpressing transgenic lines still grew well. In addition, qRT-PCR analysis revealed that the transgenic lines had significantly higher transcript levels of *PlHSP70*, which were 10-fold higher than those of the WT on average (Fig. 5d).

### Physiological indices measurement and anatomy observation of the transgenic plants

**Fig. 6:**
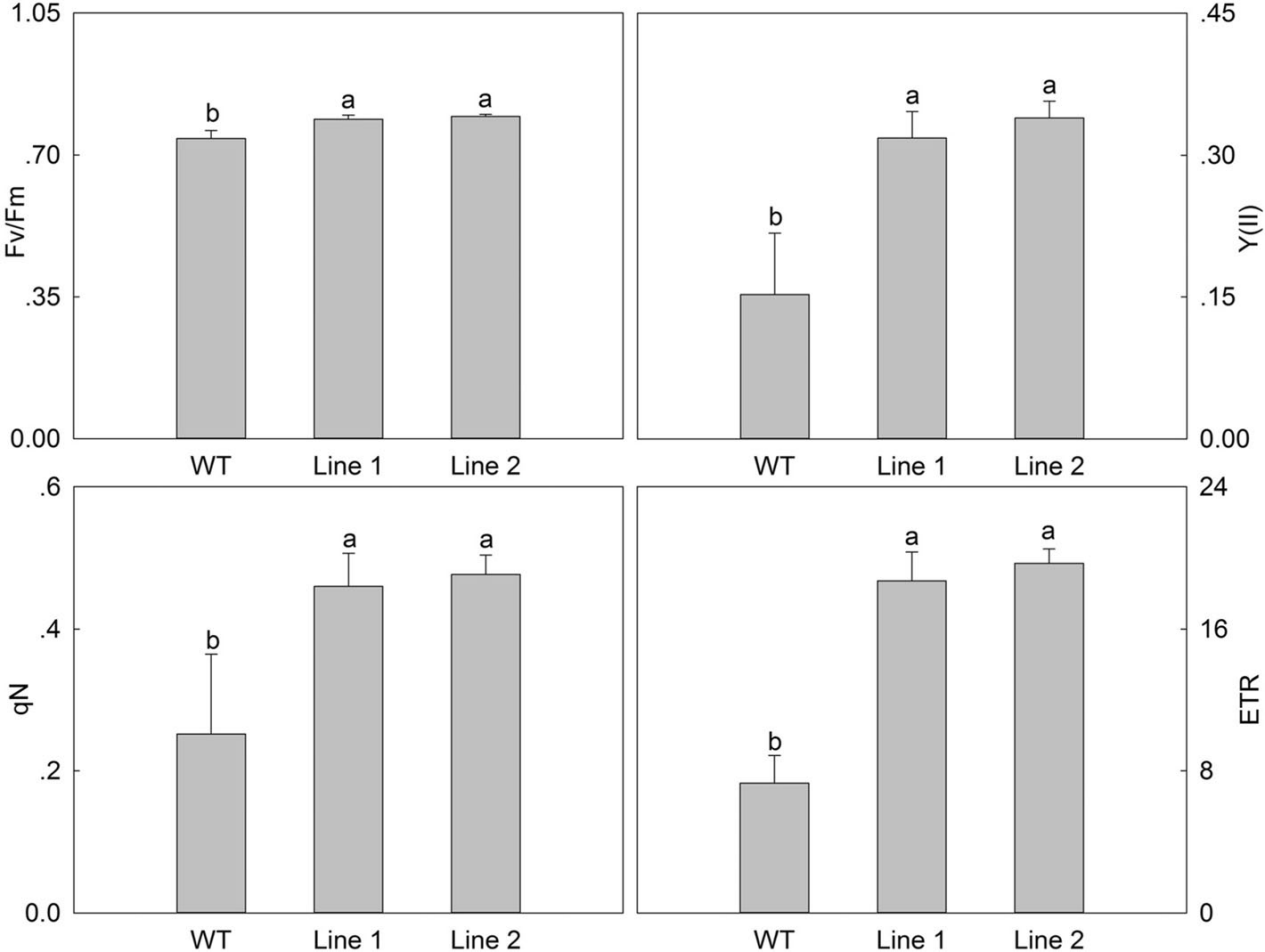
Chlorophyll fluorescence parameters of WT and transgenic lines under high temperature stress. All data are the means of three replicates with standard deviations, and different letters indicate significant differences according to Duncan’s multiple range test (*P* < 0.05)

**Fig. 7:**
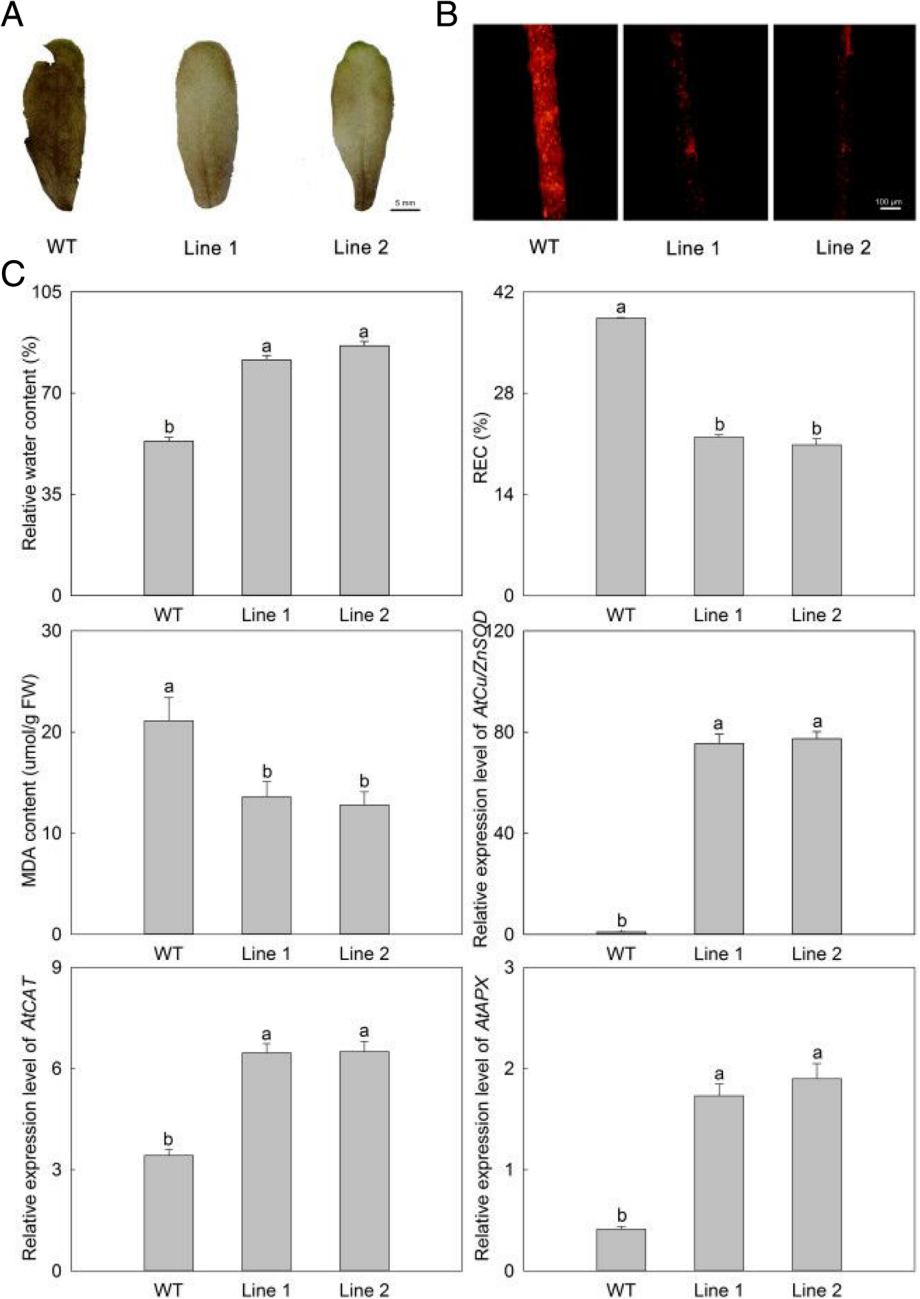
Physiological indices of WT and transgenic lines under high temperature stress. **a** H_2_O_2_ accumulation was detected by DAB staining. **b** O_2_·- accumulation was detected by a fluorescence probe. **c** Other physiological indices. All data are the means of three replicates with standard deviations, and different letters indicate significant differences according to Duncan’s multiple range test (*P* < 0.05)

**Fig. 8:**
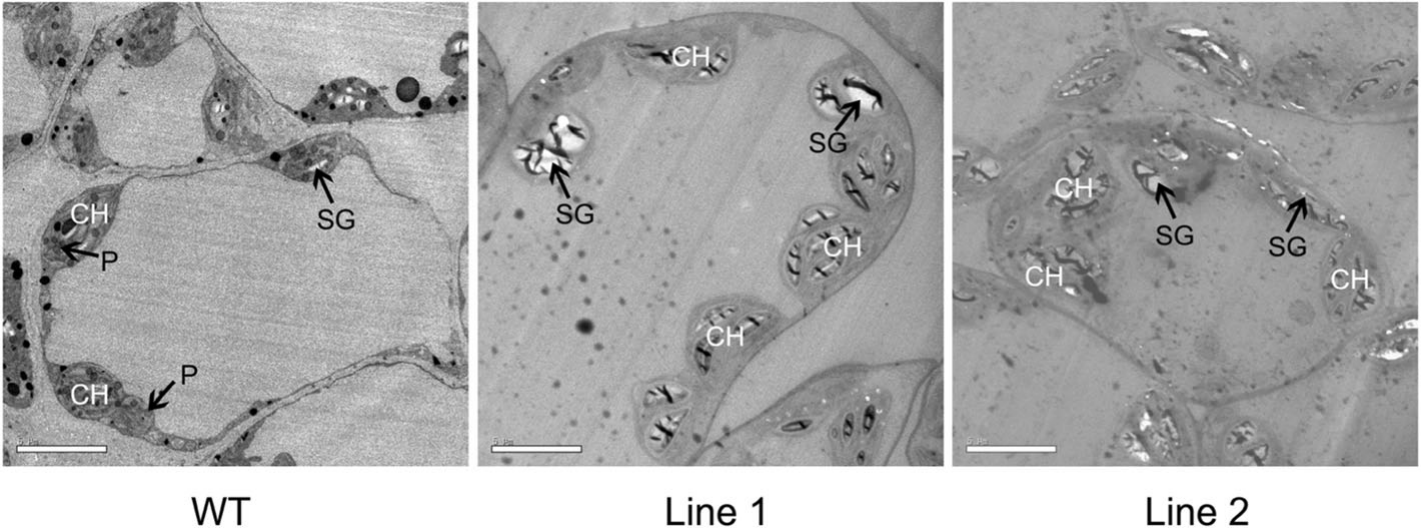
Anatomical structures of WT and transgenic lines under high temperature stress. CH: chloroplast; SG: starch grain; P: plastoglobuli

To further evaluate the growth status of the transgenic *A. thaliana* plants under high temperature stress, related physiological indices and anatomical structures were measured. Firstly, we detected the chlorophyll fluorescence parameters using a chlorophyll fluorescence spectrometer, including the ratio of variable fluorescence to maximum fluorescence (F_v_/F_m_), and the actual photosynthetic efficiency of light system II (Y (II)), non-photochemical quenching (qN) and the electron transport rate (ETR). When compared with WT, the four parameters in the *PlHSP70*-overexpressing transgenic lines were all higher, and significant differences were detected in all of the treatments; F_v_/F_m_, Y (II), qN and ETR increased by an average of 6.77, 115.45, 85.82 and 163.09%, respectively (Fig. 6). In addition, we determined the stress physiological indices including H_2_O_2_, O_2_^·-^ and REC. The H_2_O_2_ content was determined using DAB staining, and the results revealed that significant differences between WT and *PlHSP70*-overexpressing transgenic lines, and the accumulation of H_2_O_2_ decreased dramatically in the *PlHSP70*-overexpressing transgenic lines when a light color was observed compared with WT (Fig. 7a). The accumulation of O_2_^·-^ was detected using a fluorescence probe, and the results indicated that a significantly different fluorescence intensity was observed between WT and *PlHSP70*-overexpressing transgenic lines; O_2_^·-^ partially accumulated, and the fluorescence intensity of *PlHSP70*-overexpressing transgenic lines was significantly lower than that of WT (Fig. 7b). And relative water content of leaf, REC and MDA content were determined, relative water content was significantly increased in *PlHSP70*-overexpressing transgenic lines compared with WT, whereas REC and MDA content were significantly decreased in *PlHSP70*-overexpressing transgenic lines compared with WT. Then, *AtCu/ZnSOD*, *AtCAT* and *AtAPX* were significantly expressed in *PlHSP70*-overexpressing transgenic lines compared with WT (Fig. 7c). In addition, the anatomical structure of the leaves was observed. As shown in Fig. 8, broken cell membranes were found in WT, and *PlHSP70*-overexpressing transgenic lines all had intact cell membranes. Chloroplasts were the most prominent cell organelle, and they were arranged close to the cell membranes in greater quantities were mostly oval in shape. *PlHSP70*-overexpressing transgenic lines had more chloroplasts than WT. In addition, the chloroplasts of WT contained some bored plastoglobulis, whereas they were not observed in *PlHSP70*-overexpressing transgenic lines. However, a number of starch grains appeared in the chloroplasts of *PlHSP70*-overexpressing transgenic lines.

## Discussions

Numerous studies in the past years have focused on the function of *HSP70*, but its biological functions have not yet been fully elucidated. In this study, the genomic sequence of *HSP70* was isolated from *P. lactiflora* and characterized. Sequence analysis indicated that *PlHSP70* consisted of two exons and one intron. Wang et al. [27] found that the number of introns of *S. lycopersicum HSP70* ranged from 0 to 12, and most of them contained only 1 or 5–7 introns. Song et al. [28] also found that *HSP70* from *A. thaliana*, *B. campestris* and *B. rapa* all had only one intron, this finding was supported by our results. *HSP70* was part of the HSP70 family, which contained a number of highly related protein isoforms ranging in molecular weight from 66 kD to 78 kD [29]. In tree peony (*Paeonia suffruticosa* Andr.), the isolated *HSP70* cDNA encoded a 71.25-kDa polypeptide [30], and the putative molecular weight of the *PlHSP70* protein was 71.28 kDa [21]. These results revealed that the protein molecular weight of the closely related species was highly similar. And heterologous expression in this study also verified the accuracy of the previous prediction. The sequence analysis of *PlHSP70* indicated that the conserved domains play important roles in maintaining the function of HSPs, and a cytosolic compartment sequence (GPKIEEVD) at the C-terminal was found, which suggested that *PlHSP70* might be located in the cytosolic region [16]. And subcellular localization supported the prediction that *PlHSP70* was located in the cytoplasm of the cell, which was in agreement with the results from *B. campestris* [28].

Under high temperature stress, HSPs are synthesized and accumulated, and their contents are increased when the plants experience either abrupt or gradual increases in temperature [31]. In our previous studies, the expression levels of *PlHSP70* were all detected in *P. lactiflora* subjected to long-term high temperature stress, and the results showed that they all increased gradually in the early stages and then decreased [20, 21]. Whereas in this study, the potted *P. lactiflora* was treated under short-term high temperature stress, the expression level of *PlHSP70* increased gradually with the treatment, and no decline was observed. This expression pattern was consistent with the tendency of the REC, suggesting the *PlHSP70* was responsive to high temperature stress, which was in agreement with previous studies in *B. campestris* [16, 28] and soybean (*Glycine max* L.) [32]. In addition, this result reflected the fact that *P. lactiflora* might quickly perceive the changes in environmental temperature and react through the biosynthesis of large amounts of HSP70 to help it tolerate high temperature stress, and *PlHSP70* might be involved in the resistance to high temperature in *P. lactiflora*.

Several studies had reported the role of *HSP70* in response to environmental stresses. Augustine et al. [33] found that the overexpression of *Erianthus arundinaceus HSP70* increased the drought and salinity tolerance of sugarcane (*Saccharum* spp. hybrid). Transgenic expression of the *Trichoderma harzianum HSP70* increases *A. thaliana* resistance to heat, salt, osmotic and oxidative stresses [34]. *B. campestris HSP70*-overexpressing tobacco plants showed higher tolerance in response to heat stress [16]. To address the function of *PlHSP70*, it was overexpressed in *A. thaliana* under the control of the 35S CaMV promoter to evaluate its resistance to high temperature stress. And the medium screening, GUS staining and PCR of *PlHSP70* all confirmed that *PlHSP70* had been successfully transferred into *A. thaliana* plants and expressed. And the subsequent high temperature treatment showed that the overexpression of *PlHSP70* substantially enhanced the basal resistance to high temperature stress in transgenic lines compared to WT plants, and qRT-PCR analysis confirmed the significantly higher transcript levels of *PlHSP70* in transgenic lines, suggesting that *PlHSP70* might act as a molecular chaperone to confer plant high temperature tolerance.

It is clear that photoinhibition usually happens when plants are exposed to high temperature by affecting electron transport, photophosphorylation and so on, and the chlorophyll fluorescence parameters can reflect the damage of the photosynthetic reaction, including F_v_/F_m_, Y (II), qN and ETR. When *P. lactiflora* was treated under high temperature stress, these above four parameters decreased significantly [35]. In watermelon (*Citrullus lanatus* Matsum. & Nakai), F_v_/F_m_ and ETR all showed a slight drop under high temperature stress [36]. Our results revealed that F_v_/F_m_, Y (II), qN and ETR in *PlHSP70*-overexpressing transgenic lines were all significantly higher compared with WT, which suggested that photoinhibition of photosynthesis in *A. thaliana* occurred due to high temperature stress, and *PlHSP70* played an important role in alleviating the photochemical damage caused by high temperature.

High temperature can promote the accumulation of reactive oxygen species (ROS), including H_2_O_2_ and O_2_^·-^ in the chloroplasts, which induces membrane lipid peroxidation [37]. In fingered citron (*Citrus medica* var. *sarcodactylis* Swingle), high temperature caused the accumulation of H_2_O_2_ and O_2_^·-^ [38]. In this study, the accumulation of H_2_O_2_ and O_2_^·-^ were observed by DAB staining and fluorescence probe, respectively, in the leaves of *PlHSP70*-overexpressing transgenic lines and WT. However, the degree of accumulation of H_2_O_2_ and O_2_^·-^ differed drastically, with only slight accumulation in the *PlHSP70*-overexpressing transgenic lines and much greater accumulation in WT. In addition, the cell membrane damage caused by high temperature could result in the leakage of electrolytes from the cell, and REC was often considered to be an indicator for reflecting the extent of high temperature stress [39]. For example, Wan et al. [39] found that the REC of *A. thaliana* was significantly lower than that of WT under 42 °C treatment. And our study showed that *PlHSP70*-overexpressing transgenic lines had a decreased REC, suggesting *PlHSP70* played an important role in the reduction of ROS and the maintenance of membrane integrity. In addition, high temperature stress could directly destroy the cells. And among organelles, chloroplasts were the organelles most sensitive to high temperature stress and the primary location for the production of ROS [40], whose ultrastructure was inevitably affected or even damaged [10]. Chen et al. [38] observed chloroplast ultrastructural changes in *C. medica* using transmission electron microscopy and found chloroplast ultrastructural alterations, including their swelling, matrix zone expanding and lamella structure loosening. In moderately heat-tolerant *P. lactiflora*, few intact cellular structures were observed, most of the chloroplasts were swollen and assumed a near spherical shape, and their membranes were decomposed, which caused leakage of the grana lamellaes under high temperature stress [20]. In this study, *PlHSP70*-overexpressing transgenic lines had more intact cell membranes, chloroplasts and starch grains and fewer plastoglobulis than WT, which was consistent with our observation of the yellow leaves in WT. These results could provide a theoretical basis to improve the high temperature tolerance of *P. lactiflora* by genetic manipulation in the future.

## Conclusion

**Fig. 9:**
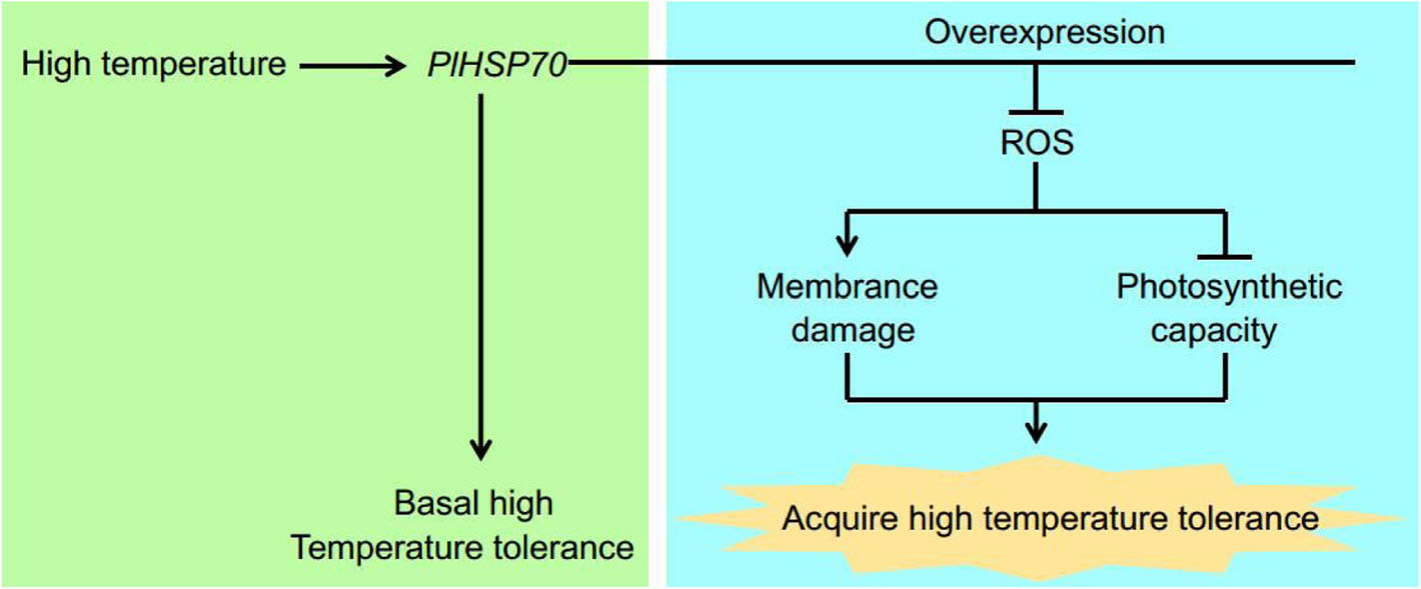
A proposed model of *PlHSP70* conferring high temperature tolerance. *PlHSP70* was involved in resistance to high temperature stress in *P. lactiflora*. The overexpression of *PlHSP70* in *A. thaliana* plants conferred high temperature tolerance by decreasing cell membrane damage and increasing photosynthetic capacity, which reduced the accumulation of ROS

In conclusion, we isolated the genomic sequence of *P. lactiflora HSP70*, which contained two exons and one intron encoding an approximately 71-kDa polypeptide that was localized in the cytoplasm of the cell. In addition, *PlHSP70* was found to be involved in high temperature tolerance in *P. lactiflora*. With relative to wild-type plants, the overexpression of this gene in *A. thaliana* decreased cell membrane damage (as indicated REC accumulation and anatomy observation) and increased the potential photosynthetic capacity (as indicated chlorophyll fluorescence parameters values) under high temperature stress by reducing the accumulation of ROS (as indicated H_2_O_2_ and O_2_^·-^ accumulation), and ultimately resulting in high temperature tolerance (Fig. 9). Thus, this study indicated that *PlHSP70* was crucial to enhance high temperature tolerance in *P. lactiflora*.

## Additional file

Additional file 1: **Table S1.** Gene-specific primers used in the gene expression analysis. (DOC 35 kb)

## Abbreviations

Amp: Ampicillin
CaMV: Cauliflower mosaic virus
cDNA: Complementary DNA
DAB: Diaminobenzidine
HSPs: Heat shock proteins
Hyg: Hygromycin
ORF: Open reading frame
PCR: Polymerase chain reaction
qRT-PCR: Quantitative real-time PCR
REC: Relative electric conductivity
ROS: Reactive oxygen species
SDS-PAGE: Sodium dodecyl sulfate polyacrylamide gel electrophoresis
sHSP: Small heat shock proteins
WT: Wild-type
