# Supplementary material for: Overexpression of herbaceous peony HSP70 confers high temperature tolerance
Source: BMC Genomics. 2019 Jan 21;20:70. doi: 10.1186/s12864-019-5448-0 (PMC6341652; doi:10.1186/s12864-019-5448-0)
Supplement: Supplementary file 1 — Table S1. Gene-specific primers used in the gene expression analysis. (DOC 35 kb) [file 12864_2019_5448_MOESM1_ESM.doc]

**Table S1. Gene-speciﬁc primers used in gene expression analysis.**

| Gene | Species | Forward primer sequence (5' - 3') | Reverse primer sequence (5' - 3') | Description |
| --- | --- | --- | --- | --- |
| *Actin* | *Paeonia lactiflora* | GTTGGGTGACACGGAAAC | ATGGCTGGAACAGGACTT | qRT-PCR |
| *HSP70* | *Paeonia lactiflora* | GAATGCTTTGGAGAACTATGCTTAC | CCACTGAATAGCCTGATCAATAGA | PCR  qRT-PCR |
| *Actin* | *Arabidopsis thaliana* | TCTCCCGCTATGTATGTCGC | TAAGGTCACGTCCAGCAAGG | PCR  qRT-PCR |
| *Cu/ZnSOD* | *Arabidopsis thaliana* | GCAGTTTTGAACAGCAGTG | TTGAAATGTGGACCAGTAGA | qRT-PCR |
| *CAT* | *Arabidopsis thaliana* | CTTGTGCTGACTTTCTCCG | AGGGTCTCTCAAGGTCTCG | qRT-PCR |
| *APX* | *Arabidopsis thaliana* | GATGCTGGAACCTATGATG | GGTCTGCGTATGTGATTTT | qRT-PCR |
